# Investing in the health workforce in Kenya: trends in size, composition and distribution from a descriptive health labour market analysis

**DOI:** 10.1136/bmjgh-2022-009748

**Published:** 2022-08-25

**Authors:** Sunny C Okoroafor, Brendan Kwesiga, Julius Ogato, Zeinab Gura, Joel Gondi, Nakato Jumba, Teresa Ogumbo, Maureen Monyoncho, Annah Wamae, Mutile Wanyee, Meldah Angir, Mona Ahmed Almudhwahi, Chagina Evalyne, Juliet Nabyonga-Orem, Adam Ahmat, Pascal Zurn, James Avoka Asamani

**Affiliations:** 1 Health Workforce Unit, Universal Health Coverage Life - Course Cluster, World Health Organization Regional Office for Africa, Brazzaville, Congo; 2 World Health Organization, Country Office for Kenya, Nairobi, Kenya; 3 Ministry of Health, Nairobi, Kenya; 4 Kenya Health Human Resource Advisory Council, Nairobi, Kenya; 5 State Department of Labour, Nairobi, Kenya; 6 Health Financing and Investment Unit, Universal Health Coverage - Life Course Cluster, World Health Organization Regional Office for Africa, Brazzaville, Republic of Congo; 7 Centre for Health Professions Education, Faculty of Health Sciences, North-West University, Potchefstroom Campus, Building PC-G16, Office 101,11 Hoffman St., Potchefstroom 2520, South Africa; 8 Health Workforce Department, World Health Organization Headquarters, Geneva, Switzerland

**Keywords:** health policy, health services research, health systems, public health, descriptive study

## Abstract

Investing in the health workforce to ensure universal access to qualified, skilled and motivated health workers is pertinent in achieving the Sustainable Development Goals (SDGs). The policy thrust in Kenya is to improve the quality of life of the population by investing to improve health service provision and achieving universal health coverage. To realise this, the Ministry of Health undertook a Health Labour Market Analysis with to generate evidence on the relationship between supply, demand and need of the health labour force. In the context of supply, Kenya has a total of 189 932 health workers in 2020 with 66% being in the public sector and 58%, 13% and 7% being nurses, clinical officers and doctors, respectively. The density of doctors, nurses and clinical officers per 10 000 in Kenya in 2020 was 30.14, which represents about 68% of the SDG index threshold of 44.5 doctors, nurses and midwives per 10 000 population. Findings indicates that Kenya needs to align future production in terms of cadre and quantity to the population health needs. Achieving this requires a multisectoral approach to ensure apposite quantity and mix of intakes into training institutions based on the health needs and ability to employ health workers produced.

**Summary box**

- Investing in the health workforce is pertinent in fostering economic growth and job creation, as well as in achieving the Sustainable Development Goals.
- Kenya’s policy thrust is to improve the quality of life of the population by investing to improve health service provision to achieve universal health coverage. To achieve this, the health sector is implementing policies aimed at planning adequately for the health workforce, and creating a favourable environment to attract and retain health workers.
- Kenya needs to invest in mechanisms to generate contextual evidence on current and future health workforce needs to guide planning at the national level and subnational levels.
- Kenya also needs to invest in the production and retention of selected cadres based on evidence on the population health needs. Achieving this requires a multisectoral approach to reach a consensus on apposite quantity and mix of intakes into training institutions, taking into account, the health needs and economic capacity of the country as well the potential for the emigration of health workers.

## Introduction

Investing in the health workforce, in development and emergency contexts, to ensure universal access to qualified, skilled and motivated health workforce, is pertinent in fostering economic growth and job creation, and in achieving the Sustainable Development Goals (SDGs).^1–3^ It is also imperative in stimulating demand for, and improving access to quality essential health services, as well as building resilience in essential health service provision to reduce the disease burdens and ensure healthy lives and wellbeing.^4^ Investing in the health workforce is critical in Africa which is plagued by a high disease burden and health workforce challenges which are hampering the achievement of national goals and the SDGs.^2 4 5^

Kenya faces a high burden of disease. Communicable diseases are the major causes of morbidity and mortality; infectious diseases and injuries contribute to more than 50% of the deaths, and non-communicable diseases contribute to approximately 39%.^6^ It is projected that in the coming years, the share of deaths from non-communicable diseases and injuries will continue to increase.^7^ As a proxy, the infant and under-5 mortality were about 39 infant deaths and 52 under-5 deaths for every 1000 live births in 2014 reducing marginally to 32 and 43 deaths per 1000 live births, respectively, in 2019.^8^ Similarly, Kenya also faces numerous health workforce challenges. Rapid devolution of health service delivery to the counties came with challenges in ensuring clarity in the roles of the national and county governments, high political influence and delays in remuneration with suboptimal motivation of health workers.^9–11^ The latter has been shown by the perennial and often prolonged health worker strikes causing an immense disruption in health services delivery.^12 13^

The policy thrust in Kenya is to improve the quality of life of the population by investing to improve health service provision and achieving universal health coverage (UHC).^14^ To achieve this, qualified, skilled and equitably distributed health workers are vital, especially at the primary healthcare level. To realise Kenya’s vision by 2030, the health sector is implementing policies that ensure adequate planning for the health workforce, and creating a favourable environment to attract and retain health workers.^15^ The sector is also implementing strategies to ensure the availability of adequate numbers, equitable distributed and accountable health workers, and financial resources are invested towards improving health workforce development and management.^15^

To achieve the 2030 target, a better understanding of the dynamics of the health labour market, and, notably, evidence of the size, composition, and distribution of health workers is needed. This is vital in ensuring that Kenya invests appropriately in its bid to ensure that its population accesses the health services it requires and UHC is attained.

Within that context, in 2021, the Government of Kenya through the Ministry of Health undertook a Health Labour Market Analysis (HLMA),^16^ an application of an economic framework for analysing the interaction and mismatches between the supply and demand for health workers and the feasibility and impact of different policy scenarios to address challenges and bottlenecks identified by the analysis.^16^ The aim of the Kenya HLMA was to generate contemporary evidence on the relationship between supply, demand and need of the health labour force in Kenya, and to develop policy actions to address the existing gaps. The Ministry of Health set up a steering committee and multistakeholder and interdisciplinary technical working groups (TWGs) with the former responsible for overall stewardship and guidance of the implementation while the TWGs being responsible for the day-to-day implementation. In this paper, we present an analysis of the key findings regarding the current situation and trends in the supply of health workers in Kenya, by describing the size, composition, and distribution of the health worker occupations in Kenya. We also present the trends in the density of health workers per population by county, the demographic distribution of health workers by sector and gender, and health professions’ education dynamics from 2016 to 2020. These findings served to answer the following policy questions that are evidence gaps in the literature: (1) What is the current stock and distribution of health workers? (2) What is the trend of the labour inflows of health workers? and (3) What is the nature of inequity in health workforce distribution?

The approach used to analyse the core aspects of the HLMA in Kenya is based on the WHO HLMA framework.^16^ Building on previous works, a multimethod approach^17^ was used to collect and analyse data on the health labour market dynamics in Kenya. These included desk review, stakeholders’ discussions (inception meetings, key informant and focus group discussions), descriptive analysis of existing quantitative data, as well as triangulation of secondary data from multiple sources such as the National Health Workforce Accounts,^18 19^ Kenya Economic Survey,^20 21^ Kenya Health Workforce Survey^22^ and annual reports of regulatory bodies. The size, composition and distribution of the health workforce in Kenya were analysed using descriptive statistics of the trends. Density is calculated per 10 000 with national and county population data obtained from the Kenya Bureau of Statistics. In this paper, we present the data and evidence obtained in the supply domain of the HLMA framework.

## Stock of health workers by occupation

As shown in table 1, Kenya has a total of 189 932 health workers across 13 major health occupations in 2020 across both public and private sectors. Of this stock, about 13 000 are doctors (including 7884 medical officers and 4908 specialists), 110 000 are nurses, 25 000 clinical officers, 1344 dentists and 987 dental technologists. Others included 1337 pharmacists, 6240 pharmaceutical technologists and 1757 physiotherapists, among others.

**Table 1:** Size and composition of health workers in Kenya, 2020

International standard classification of occupations	Occupation title in Kenya	Stock of qualified and registered	Occupation group as % share of overall stock
221—Medical doctors	Doctors	12 792	**6.70**
2211—Generalist medical practitioners	Medical officer	7884	4.20
2212—Specialist medical practitioners	Dermatologist	28	0.00
2212—Specialist medical practitioners	Obstetrician and gynaecologist	402	0.20
2212—Specialist medical practitioners	Ophthalmologist	104	0.10
2212 - Specialist medical practitioners	Paediatrician	343	0.20
2212—Specialist medical practitioners	Physician (internal medicine)	347	0.20
2212—Specialist medical practitioners	Psychiatrist	70	0.00
2212—Specialist medical practitioners	Radiologist	140	0.10
2212—Specialist medical practitioners	Surgeon	332	0.20
2212—Specialist medical practitioners	Pathologist	65	0.00
2212—Specialist medical practitioners	Anaesthesiologist	158	0.10
2212—Specialist medical practitioners	Ear, nose and throat surgeon	75	0.00
222—Nursing and Midwifery Personnel	Kenya Registered Community Health Nurse	109 659	**57.70**
2221 and 2222—Nursing and Midwifery professionals	Kenya Registered Community Health Nurse	71 539	37.70
3221—Nursing associate professionals	Kenya Enrolled Community Health Nurse	38 120	20.10
2269—Health professionals not elsewhere classified	Registered Clinical Officer	25 400	**13.40**
2269—Health professionals not elsewhere classified	Ear, Nose and Throat Clinical officer	160	0.10
2269—Health professionals not elsewhere classified	Anaesthetist Clinical Officer	932	0.50
2269—Health professionals not elsewhere classified	Lung and Skin Clinical officer	272	0.10
2269—Health professionals not elsewhere classified	Ophthalmology Clinical Officer	202	0.10
2269—Health professionals not elsewhere classified	Paediatric Clinical Officer	512	0.30
2269—Health professionals not elsewhere classified	Reproductive Health Clinical Officer	132	0.10
2261—Dentists	Dentist	1344	**0.70**
2261—Dentists	Endodontist	1	0.00
2261—Dentists	Oral and maxillofacial surgeon	29	0.00
2261—Dentists	Orthodontist	9	0.00
2261—Dentists	Periodontist	21	0.00
2261—Dentists	Prosthodontist	2	0.00
2261—Dentists	Periodontist	9	0.00
3251—Dental assistants and therapists	Dental technologist	**987**	**0.50**
2262—Pharmacists	Pharmacist	1337	**0.70**
3213—Pharmaceutical technicians and assistants	Pharmaceutical technologist	6240	**3.30**
2264—Physiotherapists	Physiotherapist	1757	**0.90**
2263—Environmental and occupational health and hygiene professionals	Occupational therapist	**553**	**0.30**
3214—Medical and dental prosthetic technicians	Orthopaedic technologist	**287**	**0.20**
2265—Dieticians and nutritionists	Nutritionist and clinical dietician	10 071	**5.30**
3212—Medical and pathology laboratory technicians	Medical laboratory technologist	10 000	**5.30**
2263—Environmental and occupational health and hygiene professionals	Public health officer	9505	**5.00**
**Total**		189 932	**100.00**

Bold text denotes main health worker categories

Fifty-eight per cent of the health workers in Kenya are nurses, and they are followed by clinical officers at 13%, doctors (7%), nutritionists and clinical dieticians and medical laboratory technologists (5.3%), and public health officers (5%), and pharmaceutical technologists (3%).

**Figure 1:**
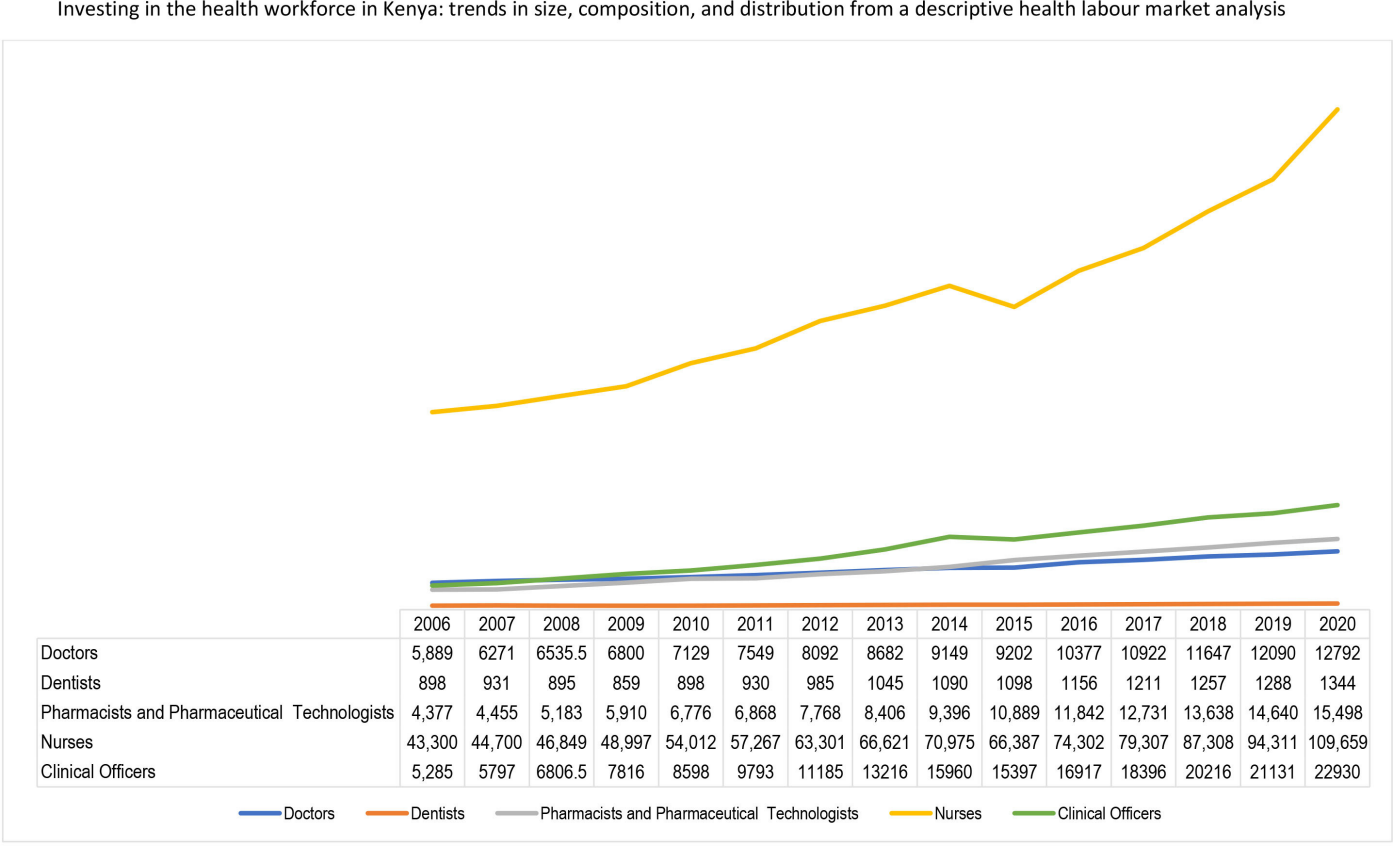
Trend in the stock of selected cadres of health workers from 2006 to 2020.

Figure 1 shows the overall stock of doctors, nurses, clinical officers, dentists, pharmacists and pharmacy technologists. The overall stock of these health professionals increased rapidly from 77 417 in 2010 to 1 62 233 in 2020. This translates to a 110% increase within a decade or approximately a 10% increase per annum. The highest growth rate within the last decade was recorded among clinical officers, increasing by 167% from 8598 in 2010 to 22 930 in 2020, with an average annual growth rate of 15%. They are followed by pharmacists and pharmaceutical technologists which increased by 129% from 6776 to 15 498, with an average annual growth rate of 129%. The stock of nurses increased by 103% and at an annual growth rate of 9% (54 012 to 109 659).

The distribution of doctors, nurses, clinical officers, and pharmacists and technologists by county is shown in table 2. Findings show an inequitable distribution of health workers by counties. For doctors, there are high proportions in Nairobi (9.3%), Kiambu (7.2%) and Machakos (5.2%), and more nurses are situated in Nairobi (6.3%), Nakuru (4%) and Kakamega (3.8%). The highest proportion of clinical officers was in Nairobi (5.8%), Migori (4%), and Kisii and Nakuru (3.3%). In comparison, these counties, respectively, have 9.3%, 2.4%, 2.7% and 4.5% of the country’s population.

**Table 2:** Distribution of selected cadres in the public sector by county in 2021

County	Population		Doctors	Nurses	Clinical officers	Pharmacists and technologists	County	Population		Doctors	Nurses	Clinical officers	Pharmacists and technologists
	Distribution	County share						Distribution	County share				
Baringo	700 516	1.4%	1.5%	1.9%	1.6%	1.6%	Marsabit	483 383	1.0%	1.0%	1.1%	1.3%	1.6%
Bomet	915 093	1.8%	0.9%	1.3%	1.4%	1.4%	Meru	1 605 096	3.2%	3.6%	3.5%	3.2%	4.5%
Bungoma	1 762 311	3.5%	1.7%	3.0%	3.0%	2.2%	Migori	1 187 418	2.4%	1.2%	2.2%	4.0%	2.1%
Busia	935 949	1.9%	2.0%	1.9%	2.2%	2.0%	Mombasa	1 266 511	2.5%	3.7%	2.9%	2.7%	3.1%
Elgeyo Marakwet	479 533	1.0%	1.0%	1.2%	1.3%	1.2%	Muranga	1 089 720	2.2%	1.8%	2.7%	2.6%	2.0%
Embu	628 637	1.3%	1.7%	2.2%	1.8%	2.3%	Nairobi	4 629 775	9.3%	8.4%	6.3%	5.8%	4.7%
Garissa	869 127	1.7%	2.4%	1.2%	1.2%	0.9%	Nakuru	2 265 829	4.6%	1.8%	4.0%	3.3%	3.4%
Homa Bay	1 187 582	2.4%	1.2%	2.5%	3.1%	2.3%	Nandi	919 865	1.8%	1.7%	1.9%	2.4%	2.1%
Isiolo	279 137	0.6%	1.2%	0.8%	0.9%	0.5%	Narok	1 225 580	2.5%	1.5%	1.4%	1.9%	2.0%
Kajiado	1 171 736	2.4%	0.6%	1.6%	2.1%	2.0%	Nyamira	632 653	1.3%	1.0%	1.9%	2.3%	3.1%
Kakamega	1 957 470	3.9%	3.1%	3.8%	3.2%	2.6%	Nyandarua	657 435	1.3%	0.7%	1.8%	0.9%	0.3%
Kericho	944 985	1.9%	3.2%	2.8%	2.1%	2.7%	Nyeri	781 990	1.6%	3.7%	2.7%	2.3%	2.2%
Kiambu	2 508 777	5.0%	7.2%	4.0%	3.0%	4.5%	Samburu	330 000	0.7%	0.7%	1.0%	0.9%	0.2%
Kilifi	1 537 730	3.1%	3.2%	2.1%	2.5%	1.4%	Siaya	1 038 223	2.1%	1.3%	2.4%	1.4%	3.0%
Kirinyaga	626 515	1.3%	1.2%	1.5%	2.0%	2.0%	Taita-Taveta	353 479	0.7%	1.4%	1.9%	2.3%	2.4%
Kisii	1 322 283	2.7%	3.8%	3.2%	3.3%	2.4%	Tana River	335 397	0.7%	0.5%	0.6%	1.2%	1.1%
Kisumu	1 211 373	2.4%	4.4%	2.3%	2.3%	3.9%	Tharaka Nithi	408 761	0.8%	1.0%	1.9%	1.9%	2.0%
Kitui	1 184 000	2.4%	3.6%	2.4%	2.6%	2.7%	Trans Nzoia	1 038 119	2.1%	1.2%	1.1%	1.7%	1.8%
Kwale	921 082	1.8%	1.9%	1.7%	2.1%	1.9%	Turkana	994 088	2.0%	1.4%	1.8%	1.7%	2.0%
Laikipia	545 338	1.1%	2.1%	1.8%	1.5%	1.7%	Uasin Gishu	1 218 837	2.4%	1.2%	1.8%	2.0%	2.1%
Lamu	150 924	0.3%	1.3%	1.5%	1.0%	1.1%	Vihiga	616 057	1.2%	0.7%	0.9%	1.2%	0.9%
Machakos	1 467 729	2.9%	5.2%	2.6%	2.7%	2.6%	Wajir	809 072	1.6%	1.5%	1.2%	0.9%	1.0%
Makueni	1 016 761	2.0%	1.8%	2.6%	1.7%	2.8%	West Pokot	669 178	1.3%	1.5%	1.7%	1.9%	2.3%
Mandera	915 829	1.8%	1.6%	1.5%	2.0%	1.7%							

## Trends in density of health workers

**Figure 2:**
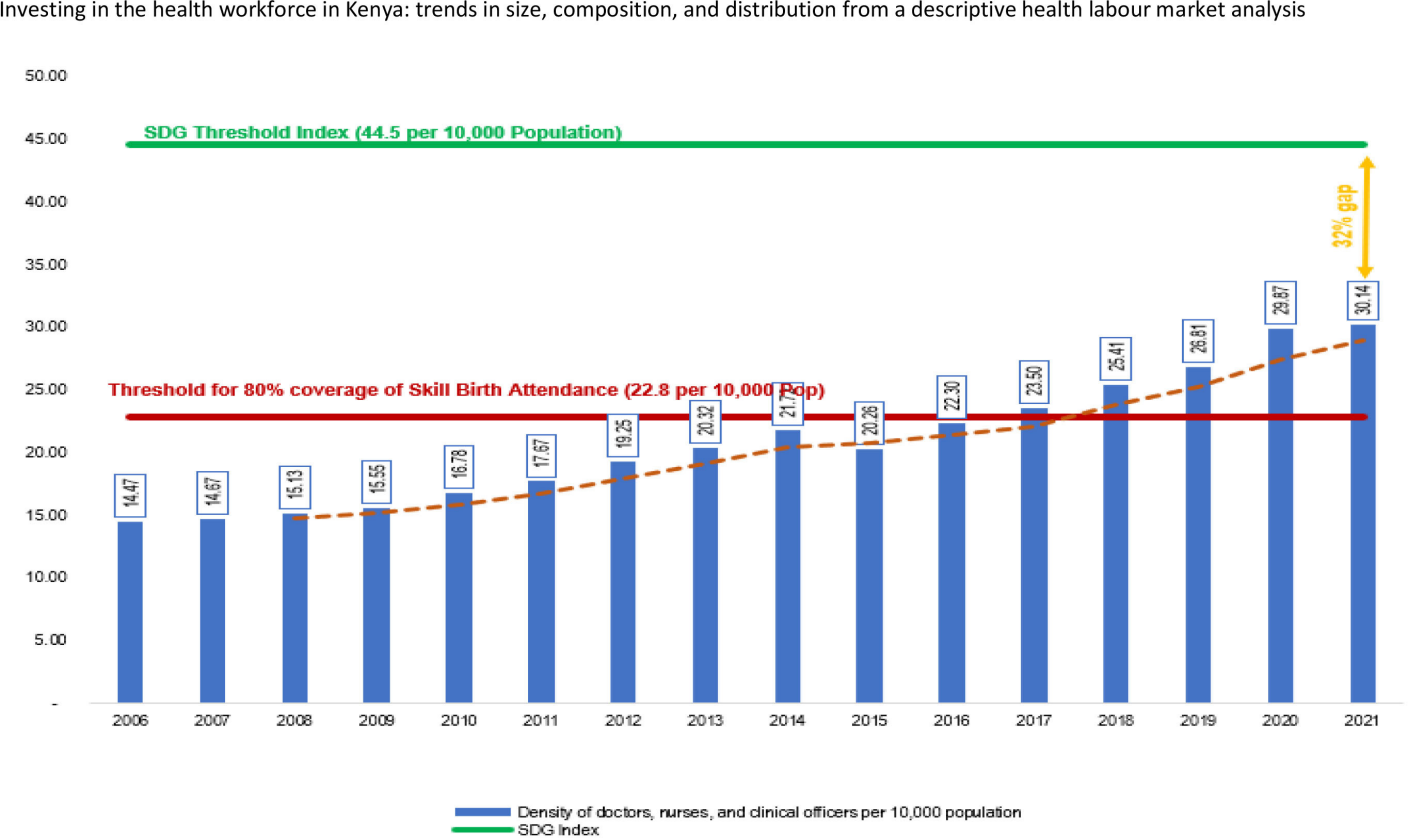
Trend in density of doctors, nurses, and clinical officers, 2006–2021. SDG, Sustainable Development Goal.

The density of doctors, nurses and clinical officers per 10 000 in Kenya in 2020 was 30.14 (figure 2). This density represents about 68% of the SDG index threshold of 44.5 doctors, nurses and midwives per 10 000 population, which is considered necessary to make good progress on the SDG 3 tracer indicators. Nevertheless, when all occupational groups are considered, the density was 38.71 health professionals and associates per 10 000 Kenyans or one health worker per 250 persons.

Figure 2 shows that the density of doctors, nurses and clinical officers per 10 000 population more than doubled between 2006 and 2021, increasing by 108% (from 14.47 in 2006 to 30.14 in 2021). The annual average annual rate of growth in the density is about 7% during the period except for 2015 when there was a decline of 6.5%, occasioned by an 18% reduction in the stock of enrolled nurses and a 3.5% decline in the stock of clinical officers. In 2016, Kenya met the 22.8 per 10 000 threshold that corresponded to 80% coverage of skilled birth attendance—a key threshold target during the millennium development goals era.^23^

**Figure 3:**
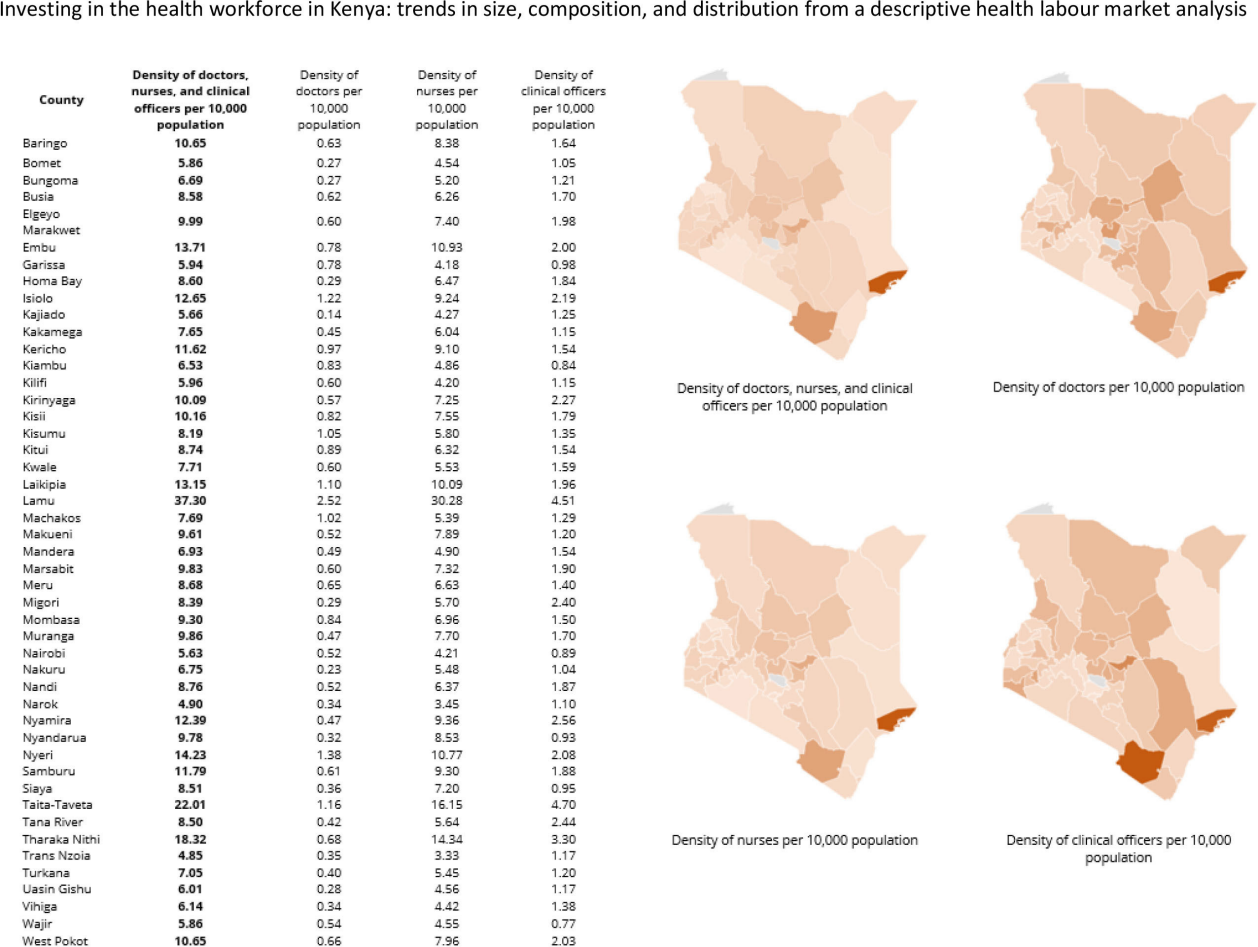
Density of doctors, nurses and clinical officers in 2020 by county.

Among the counties (figure 3), Lamu (37.30), Taita-Taveta (22.01) and Tharaka Nithi (18.32) had the highest density of doctors, nurses and clinical officers per 10 000. These counties, however, have relatively low population densities as compared with populous counties such as Nairobi, Narok and Trans Nzoia counties that had the lowest densities of 5.63, 4.90 and 4.85 health professionals per 10 000 population, respectively.

## Distribution of health workers by sector of employment and demographic characteristics

**Figure 4:**
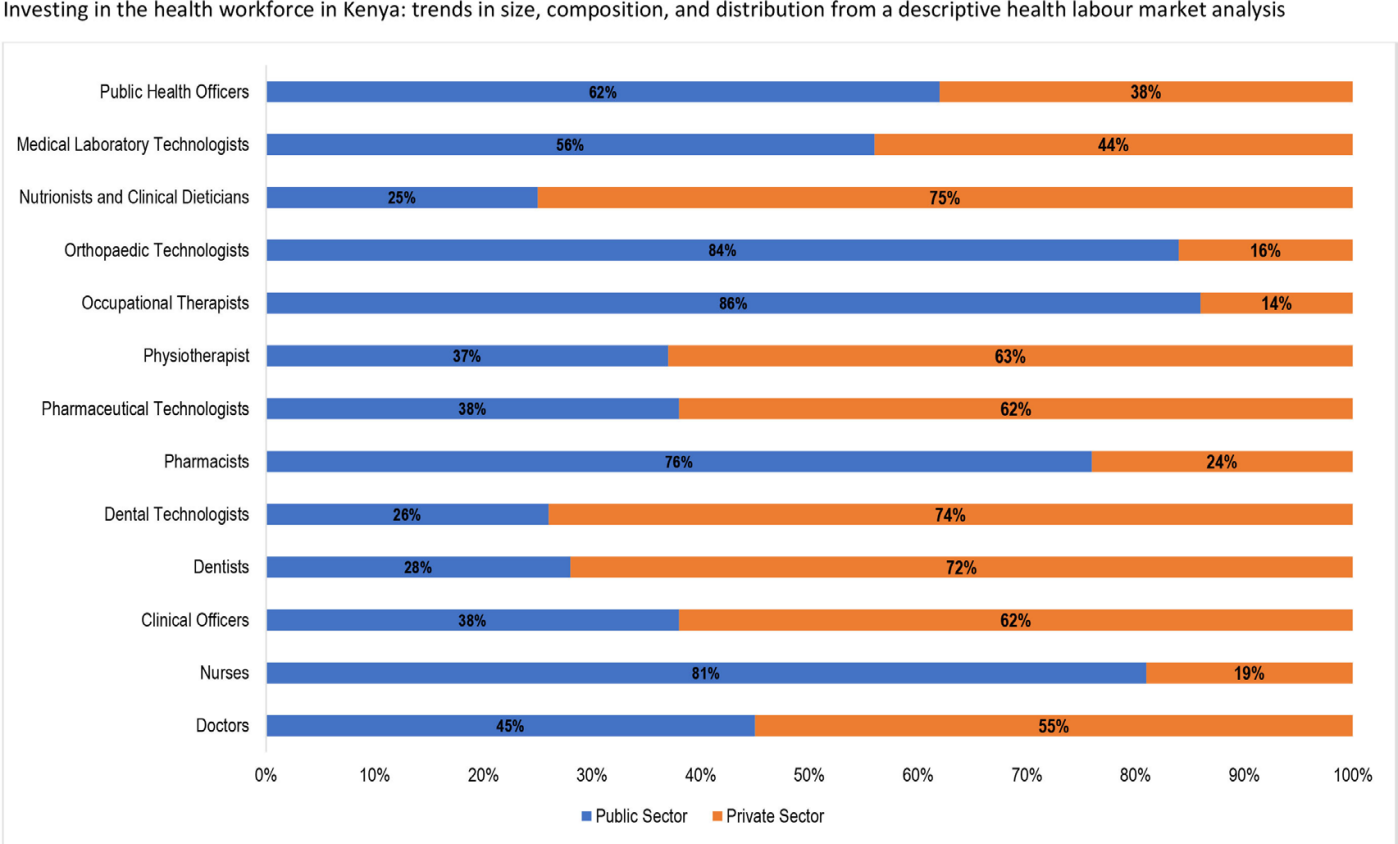
Distribution of health worker categories by sector of employment.

**Figure 5:**
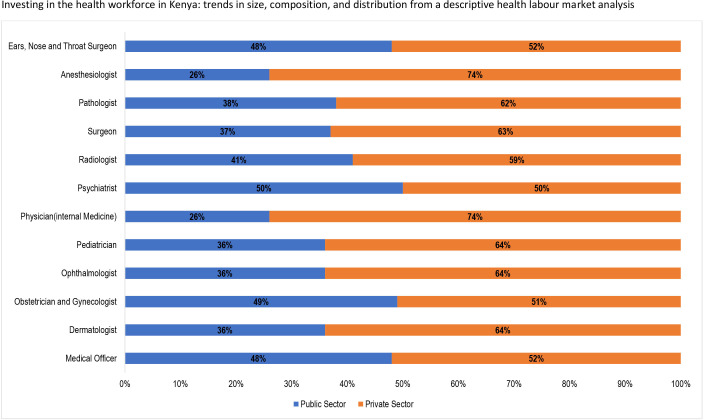
Distribution of doctors by sector of employment.

Approximately 66% of the 189 932 health workers in Kenya are employed in the public sector with the remaining 34% employed in the private sector. Still, the private sector is the main employer of doctors (55%) in comparison to the public sector’s 45% (figure 4). Figure 5 shows that approximately 62% of medical specialists are employed in the private sector, with equal proportions (50%) of psychiatrists employed in both sectors.

Eighty-one per cent of nurses that are most of the skilled health workforce in Kenya are in the public sector with the private sector absorbing only 19% of the nursing workforce (figure 4). Correspondingly, 62% of clinical officers are employed within the public sector compared with 38% in the public sector. Twenty-four per cent of the pharmacists and 62% of pharmacy technologists were in the private sector.

**Figure 6:**
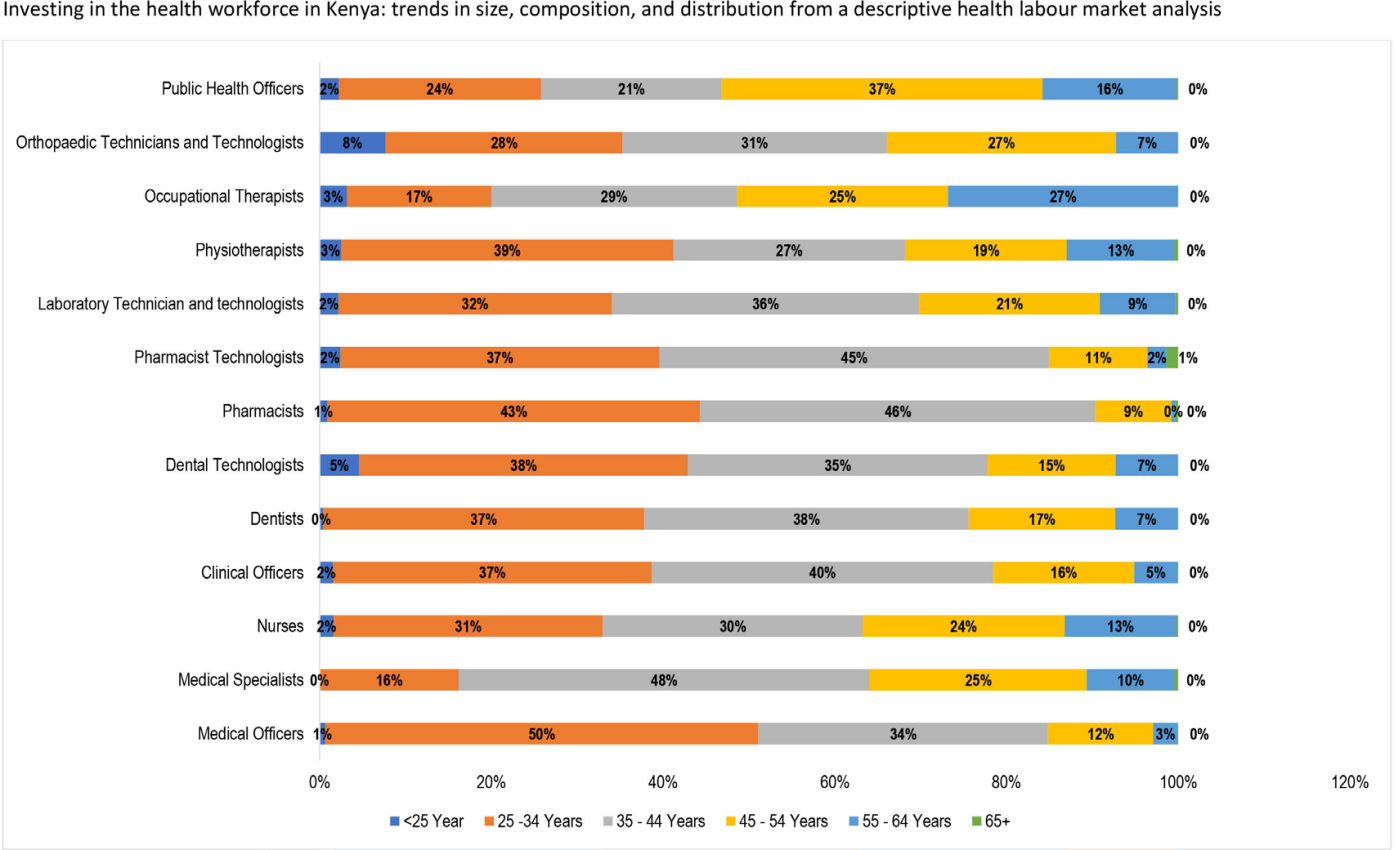
Age distribution of health workers.

Overall, 67.5% of the health professionals in the public sector are aged 25–44 years. Only 10% of these health professionals are 54 years or older, which is higher among some medical specialists—ears, nose and throat specialists (22.22%), surgeons (11.8%), and obstetrician and gynaecologist (11.73%). In particular, a little over 63% of nurses are younger than 45 years, and 70% of them are female (figure 6). Furthermore, a high proportion of medical officers (50%) are young professionals between ages 25 and 34. Majority of medical specialists (62%) are males mostly (48%) within the ages of 35–44 years old. Also, most pharmacists (46%) and pharmacy technologists (45.%) are between the age of 35–44 years, while majority of public health officers (37%) are of a higher age bracket (45–54 years).

**Figure 7:**
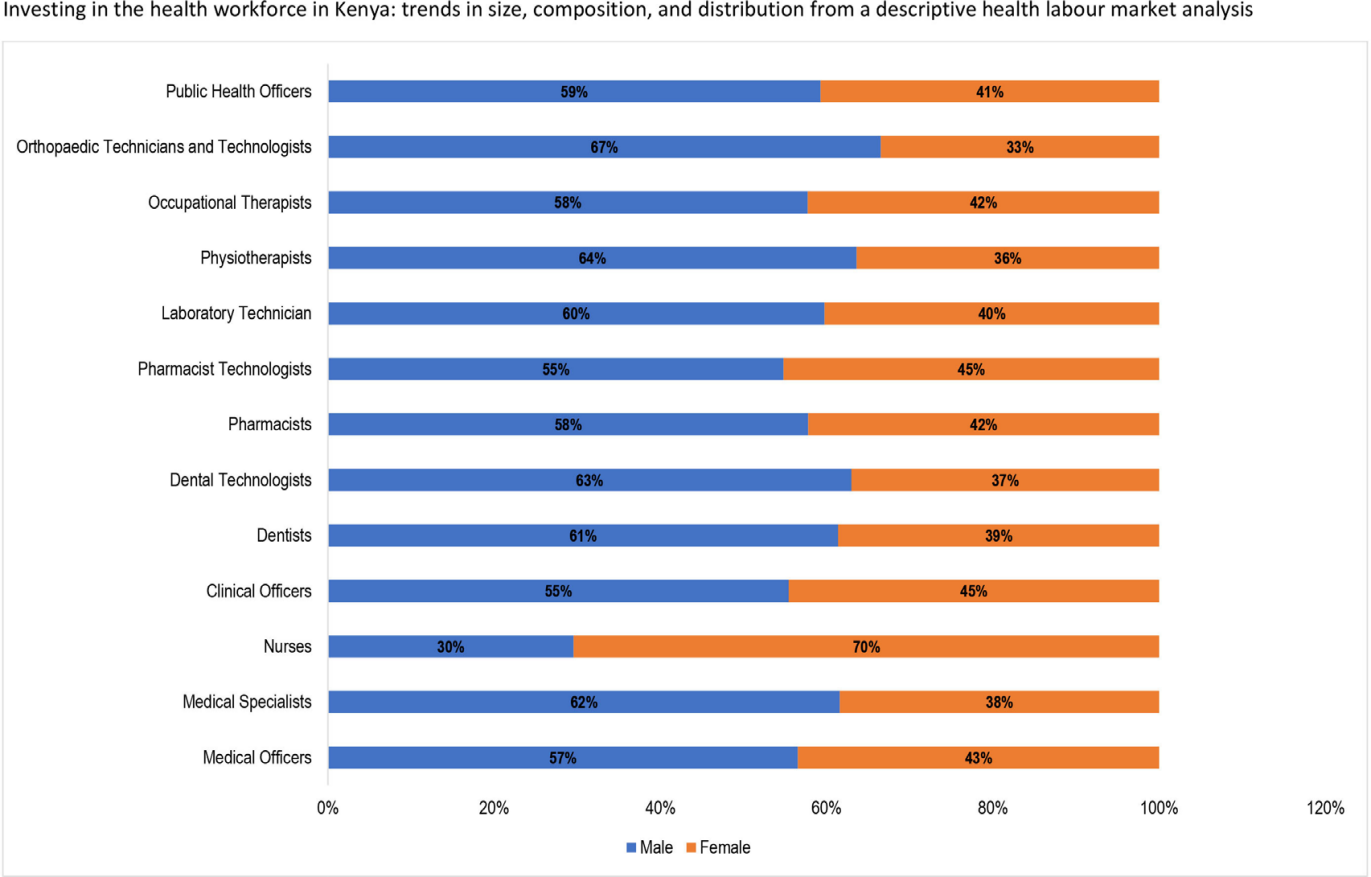
Gender composition of the health workforce.

The gender distribution of health workers in Kenya, presented in figure 7, shows that overall, 58% of the health professionals considered in this analysis are female. However, apart from nurses (70%) that have more females, most health professionals are dominated by males. Of note, there is a high proportion of orthopaedic technicians and technologists (67%), physiotherapists (64%), dentists (61%) and medical specialists (62%) are males. Other cadres that have more males than females are pharmacists technologists (55%), public health officers (59%), occupational therapists (58%), laboratory technicians and technologists (60%), pharmacists (58%) and clinical officers (55%).

## Trends in health professions education

**Figure 8:**
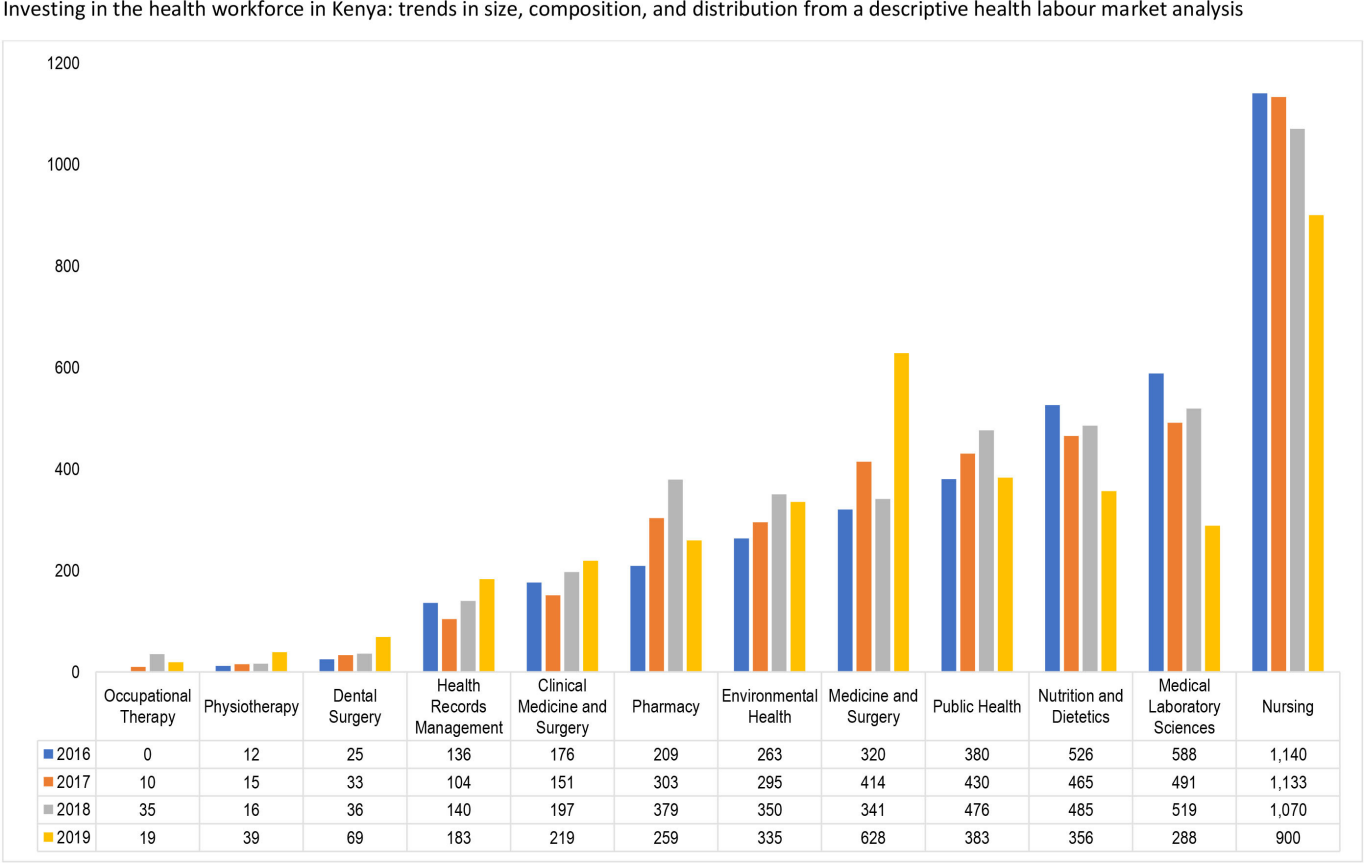
Trend in enrolments into health professions education in Kenya, 2016–2019.

The estimated annual enrolment for health profession education institutions in Kenya from 2016 to 2019 is shown in figure 8. The average highest enrolment was recorded for the nursing programmes at 904 students with the highest number of 1140 students enrolled in 2016 and the lowest number of 900 students in 2019 An average of 341 students were enrolled in medicine and surgery programmes, with 628 students enrolled in 2019. Three programmes, occupational therapy, physiotherapy and dental surgery enrolled less than 50 students from 2016 to 2019, with health records management and clinical medicine and surgery programmes (for clinical officers) enrolling an average of 113 and 149 students, respectively.

**Figure 9:**
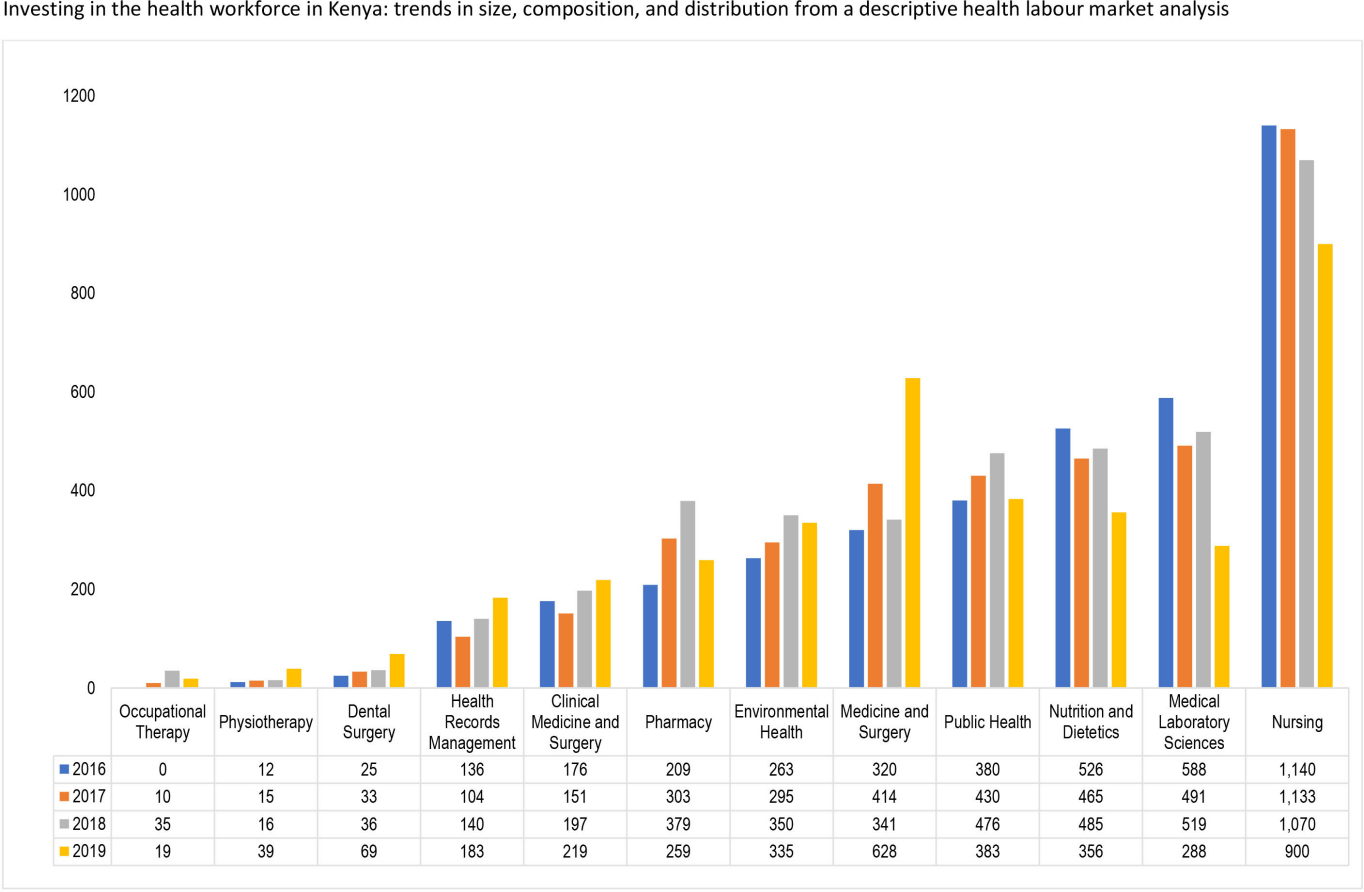
Trend in graduates into health professions in Kenya, 2016–2019.

Figure 9 indicates that the number of graduates from the medicine and surgery programme increased by 96% between 2016 and 2019 from 320 to 628 graduates. The number of graduates from the pharmacy programme increased by 84% from 209 in 2016 to 379 graduates in 2018, before declining to 259 in 2019 (a decline of 31.7% from the 2018 level). For the nursing programmes, the number of graduates declined consistently by 21% from 1140 in 2016 to 900 in 2019. The biggest decline was, however, recorded by the medical laboratory sciences programme where in 2016 the number of graduates was 588, which declined by more than 49% to 288 graduates in 2019.

## Implications for investing in the health workforce in Kenya

The urgent need for countries to ensure equitable access to health workers within enhanced health systems; towards achieving national goals, UHC the SDG has been advocated.^1 2^ While policy-makers and health managers in several countries strive to achieve this, access to essential information on the health workforce has remained a long-standing challenge.^18^ The absence of this critical information has impeded apposite planning for the health sector and the health workforce. Hence, the chronic health workforce issues including weak HRH leadership, governance and stewardship, poor motivation and retention strategies, high migration rate, low production rate, lack of contemporary HRH evidence, and poor remuneration packages have remained evident.^2 24^ Therefore, building a robust mechanism for ensuring reliable and comprehensive HRH information is critical as countries, including Kenya, make progress towards strengthening the health system and ensuring equitable distribution of the health workforce. Generating, analysing and disseminating HRH data and evidence at the national and county level in Kenya will ultimately promote problem-based planning and catalyse the achievement of national goals, UHC and the SDG.

The overall stock of doctors, nurses, clinical officers, dentists, pharmacists and pharmacy technologists in Kenya increased by 110% from 2010 to 2020—approximately a 10% increase per annum. Informed by Kenya’s drive to urgently expand primary healthcare and fill in staffing gaps resulting from critical shortages of doctors amidst the country’s commitment to the attainment of key health targets, the country invested in producing the various cadres by scaling up training institutions across the counties. The advent of devolution was also associated with heavy investment in recruitment of health workers. This has boosted access to health services, culminating in improved UHC coverage index from 30 in 2000 to 56 in 2019.^25^

The density of health workers per population indicates the availability of health workers to provide services. The density of doctors, nurses and clinical officers per 10 000 population in Kenya doubled by 108% from 14.47 in 2006 to 30.14 in 2021 from 2006 to 2021 with the average growth rate being 7%. Cumulatively, Kenya still has a 32% gap if it is to achieve the 4.45 per 1000 SDG threshold index. Therefore, this study clearly exposes the need for Kenya to consciously invest in policies and plans towards improving the availability of an active health workforce if it will close this gap and ensure equitable distribution of the density per population at national level and by counties.

Achieving the above requires a substantial investment in contextual evidence generation based on the health labour market dynamics.^26^ Studies should focus on subnational evidence generation on the impact of decentralisation modalities on the supply (education) and demand (remuneration, policy environment and strategies for attraction and retention). Such studies potentially unravel the effect of complex interlinked factors that need to be addressed to improve the availability of health workers.^10 17 27–32^

Analysis of the public–private employment of health workers in Kenya indicates that 66% were employed in the public sector as was also reported in a previous HLMA in Kenya.^33^ For the cadres, high proportions of nurses and clinical officers are employed within the public sector. The private sector employed higher proportions of doctors, pharmacists and pharmacy technologists. This situation indicates the potential of a shortage of skilled health workers and inadequate skill mix in some counties and rural and remote areas in Kenya.^33 34^ The findings indicate a key role of the private sector in Kenya’s health system. An in-depth review of the private sector contribution in the health system of Kenya is worth exploring.

Enrolment and graduation from health professions education in Kenya indicate that nursing is a course of choice, and it is closely followed by medical laboratory sciences nutrition and dietetics and public health. A previous study showed that the trends in graduates of nurses from 2005 to 2010 ranged from 1676 in 2005 to 3879 in 2009,^33^ while our findings indicates that from 2016 to 2019, the graduates ranged from 900 to 1140. This suggests that the production of nurses has also reduced over the years despite it still being the most attractive. As mentioned previously, studies on the interests attracting more students to these programmes are needed to make other programmes attractive. An interesting finding is that the cumulative enrolment into medicine and surgery is higher than for clinical medicine and surgery. This indicates that government needs to plan to attract and retain these health workers to ensure a more even distribution of doctors across the country as it strives to improve equitable access to health workers.

Our study had limitations. We used data available in the Kenya Economic survey and the NHWA. Both sources used information obtained from various sources in the country. While the information on the trend and size of health workers in Kenya provides useful insight, it should be interpreted with caution. This is because most of the reported professionals may be part of the active health workforce since the migration rates are not tracked and reported in the NHWA^34 35^ and Kenya Economic Surveys. Additionally, there is a strong likelihood that updating of the registries where the information was obtained may not be regular and the reported size of health workers may not be very accurate. Additionally, none of the data sources reported the dual practice to allow for a clear delineation of health workers that work in the public and private sectors as well as in rural and urban areas concurrently. This has been identified as a gap in health workforce information.^34^

Despite these limitations, the findings highlight some clear areas that Kenya needs to invest in as it strives to achieve UHC and the SDG. There is a need to invest in mechanisms to generate contextual evidence on current and future health workforce needs. This should focus on the national level and subnational levels to ensure comprehensive evidence is generated for planning at all levels. Complimentarily, health workforce information systems need to be strengthened to ensure the ready availability of data for planning and monitoring. Kenya also needs to align future production in terms of cadre and quantity to the population health needs of the country. Evidence on the population’s health needs will inform this and guide investments in the cadres for focus. Achieving this requires a multisectoral approach to ensure that consensus is reached on apposite quantity and mix of intakes into training institutions, taking into account, the health needs and economic capacity of the country as well the potential for the emigration of health workers. Participation should include but not be limited to Ministries of Health, Finance, Education, Labour, private sector, Public Service Commission, health training institutions and development partners.

This analysis focuses on the supply component of the health labour market framework. Other components of the framework, specifically macroeconomics, demand and need requires further exploration. Evidence on these should focus on understanding the demand dynamics, predicting the normative need and policy actions needed to address the gaps in demand that is required to achieve the desired need. Analysis on these is recommended for future research.

## Conclusion

For Kenya to achieve its policy drive of improving the quality of life of the population by improving health service provision to achieve UHC, there is an urgent need to invest in the health workforce. This is informed by the 32% gap to attain the 4.45 per 1000 SDG threshold index, and the low number of specialised workforce as well as the high proportion of current health workers in their 50s—thus the need to increase stock focusing in the younger age groups to ensure continuity of health service provision. Also, some cadres are much lower than others and this impedes efforts to deliver integrated people-centred health services. Investing in the health workforce requires a multisectoral approach to health workforce planning and management. Equally important is evidence on population health needs, which should guide the implementation of strategies directed at ensuring that all Kenyans have access to a qualified, skilled, motivated and equitably distributed health workforce.

## Data Availability

No data are available.
